# Miracle Fruit (*Synsepalum dulcificum*) Exhibits as a Novel Anti-Hyperuricaemia Agent

**DOI:** 10.3390/molecules21020140

**Published:** 2016-01-26

**Authors:** Yeu-Ching Shi, Kai-Sian Lin, Yi-Fen Jhai, Bao-Hong Lee, Yifan Han, Zhibin Cui, Wei-Hsuan Hsu, She-Ching Wu

**Affiliations:** 1 Taiwan Indigena Botanica Co., Ltd., Taipei 11458, Taiwan; jasmineycs@yahoo.com.tw; 2 Department of Food Sciences, National Chiayi University, Chiayi 60004, Taiwan; Tracy12095238@hotmail.com (K.-S.L.); even78819@gmail.com (Y.-F.J.); 3 Division of Hematology and Oncology, Department of Internal Medicine, Taipei Medical University Hospital, Taipei 11042, Taiwan; f96b47117@ntu.edu.tw; 4 Department of Traditional Chinese Medicine, Taipei Medical University Hospital, Taipei 10042, Taiwan; 5 Department of Oral Pathology, Ninth People’s Hospital, Shanghai Jiao Tong University School of Medicine, Shanghai 200092, China; yifanhan2014@gmail.com; 6 Department of Comparative Pathobiology, Purdue University, West Lafayette, IN 47907, USA; cuizhibin1985@gmail.com; 7 Biochemical Process Technology Department, Center of Excellence for Drug Development, Biomedical Technology and Device Research Laboratories, Industrial Technology Research Institute, Hsinchu 30058, Taiwan

**Keywords:** miracle fruit, xanthine oxidase, monosodium urate (MSU), oxonic acid potassium salt, anti-hyperuricaemia agent

## Abstract

Miracle fruit (*Synsepalum dulcificum*) belongs to the Sapotaceae family. It can change flavors on taste buds, transforming acidic tastes to sweet. We evaluated various miracle fruit extracts, including water, butanol, ethyl acetate (EA), and hexane fractions, to determine its antioxidant effects. These extracts isolated from miracle fruit exerted potential for reduction of uric acid and inhibited xanthine oxidase activity *in vitro* and in monosodiumurate (MSU)-treated RAW264.7 macrophages. Moreover, we also found that the butanol extracts of miracle fruit attenuated oxonic acid potassium salt-induced hyperuricaemia in ICR mice by lowering serum uric acid levels and activating hepatic xanthine oxidase. These effects were equal to those of allopurinol, suggesting that the butanol extract of miracle fruit could be developed as a novel anti-hyperuricaemia agent or health food.

## 1. Introduction

Miracle berry (*Synsepalum dulcificum*) is also called miracle fruit. It has a large seed that is surrounded by a thin layer of berry flesh with a faint cherry-like flavor [1]. There is growing interest in the potential use of miracle berries in food, as it has the unique ability to make sour foods taste sweet. This effect lasts until miraculin is diluted and eliminated by saliva. Miracle fruit is reported to have antioxidant activities [2] and improves the sweetness of low-calorie desserts without increasing energy compensation. Thus, miracle fruit could represent a novel sweetener for use in food [3].

Gout is an inflammatory disease caused by the over-production of uric acid in the blood and crystallization of monosodiumurate (MSU) in tissues. This process is driven by neutrophil influx into joints, leading to acute inflammatory arthritis with severe pain in the affected tissue. MSU crystals can trigger interleukin-1β maturation via recruitment of a cytosolic complex, called the nucleotide-binding oligomerization domain-like receptor pyrindomain containing 3 (NLRP3) inflammasome [4,5]. Redox signalling molecules, such as reactive oxygen species (ROS), are generated by NLRP3 inflammasome activators, including MSU and other endogenous danger signals [6].

Another potential source of cellular ROS is xanthine oxidase; however, its role remains unclear. Xanthine oxidase is a key enzyme in purine catabolism, mediating the formation of uric acid, which can be further broken down to allantoin in mammals that possess uric oxidase (uricase) [7]. Interestingly, several clinical and experimental studies suggest that xanthine oxidase activity has pro-inflammatory effects [8]. Currently, numerous anti-gout agents are available, including non-steroidal anti-inflammatory drugs, such as indomethacin and naproxen. They are frequently used as first-line therapies for acute gout. Nevertheless, their use is limited by adverse reactions, including gastrointestinal toxicity, renal toxicity, and gastrointestinal bleeding. Therefore, the identification of better anti-gout arthritis drugs is necessary.

We hypothesized that the anti-gouty arthritis effects of miracle fruit may be mediated by inhibition of inflammatory cell activation and enhanced antioxidant activity. We evaluated the anti-inflammatory effect of miracle fruit in MSU crystal-treated RAW264.7 macrophages and investigated miracle fruit-induced attenuation of xanthine oxidase activity in animal models. Our results may support the utility of miracle fruit in herbal medicines.

## 2. Results and Discussion

### 2.1. Antioxidant Effect and Xanthine Oxidase Suppression Following Miracle Fruit Treatment in Vitro

Hyperuricaemia results from increased uric acid production impaired renal uric acid excretion. In most patients with primary gout, hyperuricaemia results from inefficient renal excretion. However, in approximately 10% of cases, hyperuricaemia is due to endogenous uric acid overproduction [9]. Hyperuricaemia is associated with inflammation, mediated by ROS and cytokine generation through inflammasome activation [10]. Furthermore, MSU is reported to trigger oxidative stress and inflammasome activity [6]. MSU is known to activate the inflammasome, thereby triggering oxidative stress and hyperuricaemic-like inflammation in monocytes or macrophages [7]. Antioxidants can have protective effects by blocking MSU-induced inflammasome activation *in vitro* and *in vivo* [7,11]. Recent work has shown that miracle fruit has antioxidant properties [2].

**Figure 1:**
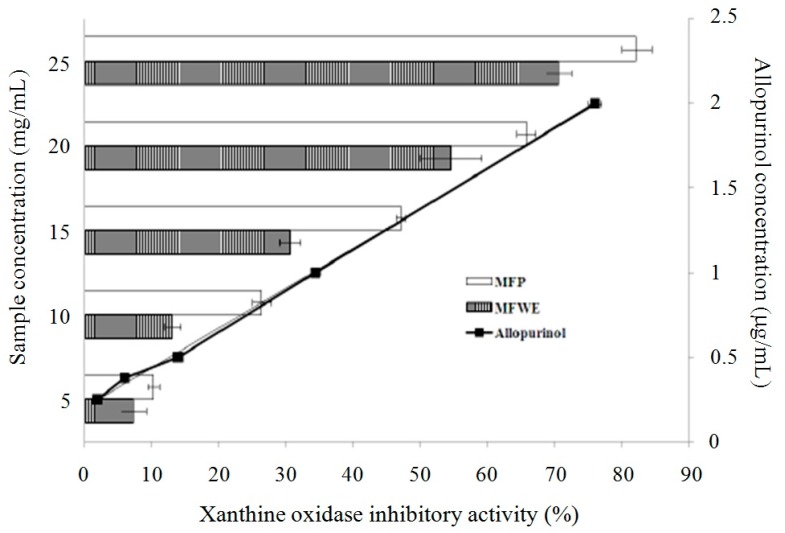
The effects of mircle fruit powder (MFP) and miracle fruit-water extract (MFWE) on xanthine oxides activity *in vitro*. Allopurinol: positive control. Each value is expressed as mean ± S.D. (*n* = 3).

**Figure 2:**
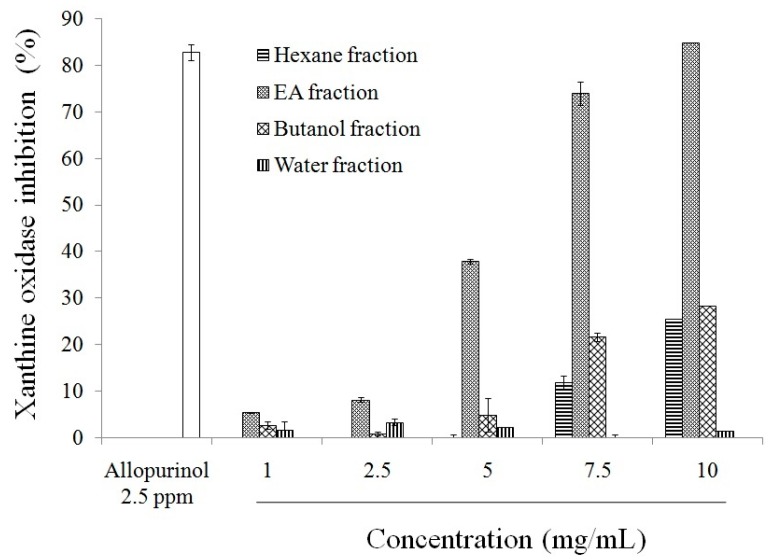
Xanthine oxidase inhibitory activities of different solvent fractions from butanol extract of miracle fruit. Each value is expressed as mean ± S.D. (*n* = 3). EA: ethyl acetate.

**Figure 3:**
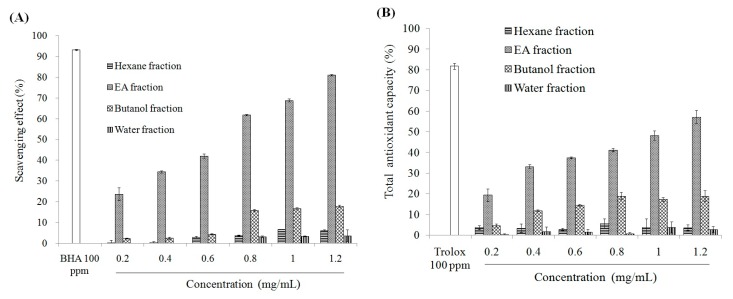

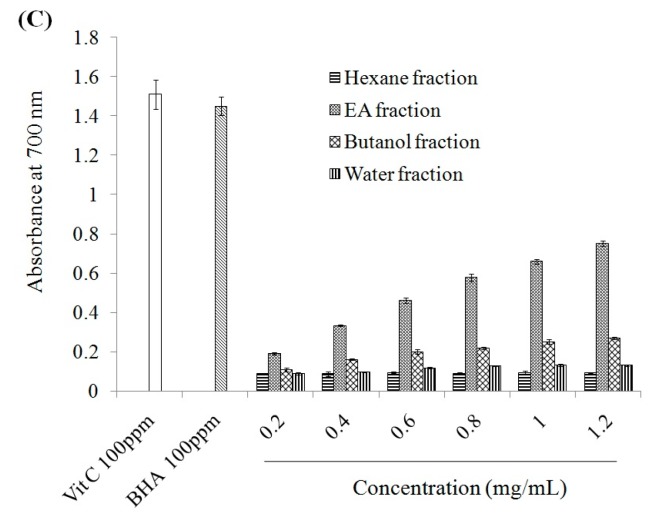
The effects of different solvent fractions from butanol extract of miracle fruit on (**A**) DPPH radical scavenging capacity; (**B**) Trolox Equivalent Antioxidant Capacity (TEAC) activity; and (**C**) reducing power. Each value is expressed as mean ± S.D. (*n* = 3). EA: ethyl acetate.

As shown in Supplemental Table S1, miracle fruit powder (MFP) has more total phenolic compounds, flavonoids, and anthocyanins than miracle fruit water extract (MFWE). Therefore, MFP exerted greater DPPH and ABTS radicals scavenging activity, and reduced power, as shown in Supplemental Figure S1. Additionally, MFP and MFWE inhibited xanthine oxidase activity (Figure 1). However, MFWE had a dose-dependent effect on xanthine oxidase activity *in vitro* (Supplemental Figure S2A). We also found that MFWE markedly suppressed xanthine oxidase activity in MSU-treated RAW264.7 macrophages, with 500 μg/mL MFWE exerting a greater effect than 100 μg/mL allopurinol (Supplemental Figure S2B). MFP was partitioned using water, butanol, ethyl acetate (EA), and hexane after extraction with butanol. Total phenolic compounds and flavonoids were measured in these extracts. Our study revealed that the EA fraction contains high levels of phenolic compounds (mg·GAE/g) and flavonoids (5.38 mg·QE/g) (Table 1). The EA fraction exhibited greater xanthine oxidase inhibition than the water, butanol, and hexane fractions (Figure 2). Thus, among the miracle fruit fractions, the EA fraction may exert anti-oxidant activity, including dose-dependent DPPH scavenging activity, ABTS scavenging activity, and power reduction (Figure 3).

**Table 1:** Total phenolics and flavonoids of different solvent fractions from butanol extracts of miracle fruit.

Fractions	Total Phenolics	Flavonoids
Water Fraction	0.91 ± 0.16 ^c^	1.12 ± 0.14 ^b^
Butanol Fraction	4.15 ± 0.36 ^b^	1.28 ± 0.13 ^b^
EA Fraction	12.91 ± 1.15 ^a^	5.38 ± 0.52 ^a^
Hexane Fraction	1.03 ± 0.99 ^c^	1.32 ± 0.17 ^b^

Each value is expressed as mean ± S.D. (*n* = 3). Data bearing different superscript letters (a–c) are significantly different (*p* < 0.05). Total phenolics were accorded to gallic acid equivalent (GAE); flavonoids were accorded to quercetin equivalent (QE).

### 2.2. Inhibition of Oxidative Stress in MSU-Treated RAW264.7 Macrophages

**Figure 4:**
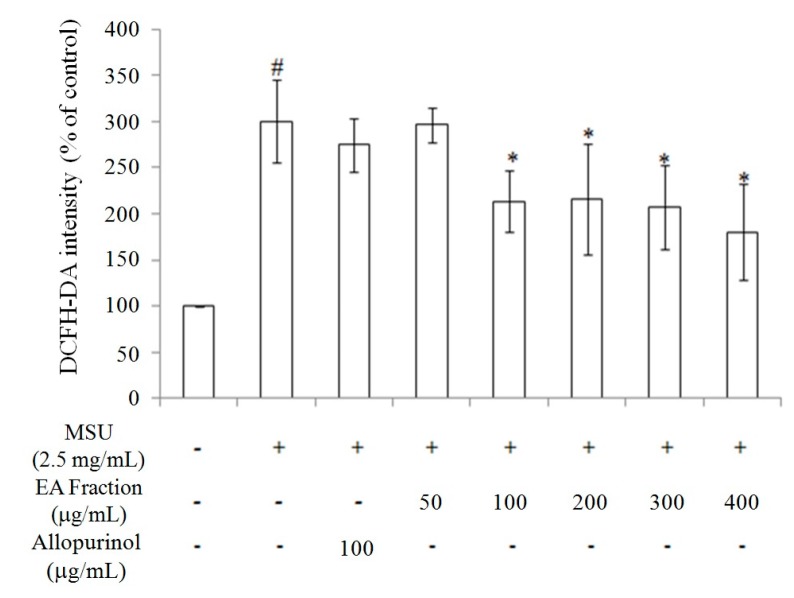
The inhibitory effects of the ethyl acetate (EA) fraction from butanol extract of miracle fruit on reactive oxygen species production in MSU crystal-induced in RAW264.7 cells. Each value is expressed as mean ± S.D. (*n* = 3). Data bearing different superscript letters (# and *) are significantly different (*p* < 0.05). # Compare with control; * Compare with only MSU. MSU: monosodium urate crystals.

Because the EA fraction had the strongest antioxidant effect and inhibited xanthine oxidase activity, we evaluated the effect of the EA fraction on reactive oxygen species (ROS) generation in RAW264.7 macrophage treated by MSU (2.5 mg/mL) for 24 h. As shown in Figure 4, the EA fraction (100–400 μg/mL) inhibited ROS generation in MSU-treated RAW264.7 macrophages. Furthermore, 100 μg/mL EA fraction suppressed ROS generation to a similar extent as 100 μg/mL allopurinol. These findings indicate that the anti-uric acid effect of allopurinol occurred independently of oxidative stress inhibition. Taken together, we have shown that the butanol extracts and various miracle fruit fractions, including water, butanol, EA, and hexane fractions, exert antioxidant activity, thereby suppressing ROS generation (Figure 3 and Figure 4; Supplemental Figure S2; Table 1; Supplemental Table S1).

### 2.3. Miracle Fruit Downregulates Uric Acid Levels in Hyperuricaemic Mice

Xanthine oxidase catalyses hypoxanthine and xanthine oxidation, leading to uric acid formation [12]. Therefore, xanthine oxidase inhibitors may represent potential therapeutic agents to treat hyperuricaemia, as they could be used to block uric acid biosynthesis [13]. Although several medicinal plants are used to prevent and treat hyperuricaemia and gout, based on traditional medicine systems [14,15], use of miracle fruit to treat hyperuricaemic is minimal, due to a lack of formal scientific evidence to support its effectiveness. Miracle fruit contains epicatechin, rutin, quercetin, myricetin, kaempferol, gallic acid, ferulic acid, syringic acid, three anthocyanins (delphinidin glucoside, cyanidin galactoside, and malvidin galactoside), three tocopherols, and lutein [2,16].

The anti-hyperuricaemic effects of miracle fruit were investigated *in vivo* using ICR mice treated with oxonic acid potassium salt (250 mg/kg·bw) for seven days. During the induction period, the mice were orally administered low dose miracle fruit butanol extract (MFL; 500 mg/kg·bw/day), high dose miracle fruit butanol extract (HFL; 1000 mg/kg·bw/day), or allopurinol (10 mg/kg·bw/day; positive control). Allopurinol is the most commonly used xanthine oxidase inhibitor and is prescribed clinically for gout treatment. However, its use is limited by hypersensitivity, Stevens–Johnson syndrome, renal toxicity, and fatal liver necrosis [17]. Attempts have been made to identify natural compounds that inhibit xanthine oxidase and have fewer side effects, thereby serving as allopurinol [10,18]. Our results suggest that butanol extracts from miracle fruit do not induce side effects in organs (Table 2) or alter serum biochemical parameters in oxonic acid potassium salt-treated ICR mice (Table 3). No significant difference was observed in organs, including the liver and kidneys (Table 2). Additionally, serum creatinine (CRE) and blood urea nitrogen (BUN) levels were unchanged in oxonic acid potassium salt-induced ICR mice, suggesting that there were no toxic effects. Furthermore, no side effects were observed (Table 3).

**Table 2:** Effect of butanol extract from miracle fruit on relative liver and kidney weight of hyperuricemia mice.

Groups	Relative Organ Weight (g/100 g of bw)	
	Liver	Kidney
Normal	4.84 ± 0.51	1.57 ± 0.14
NC	5.12 ± 0.62	1.58 ± 0.19
PC	4.96 ± 0.36	1.59 ± 0.14
MFL	5.01 ± 0.51	1.61 ± 0.16
MFH	5.22 ± 0.28	1.67 ± 0.2

Each value is expressed as mean ± S.D. (*n* = 5). Value in a column with the different superscripts are significantly different (*p* < 0.05). Normal: blank, NC: negative control, PC: positive control (allopurinol, 10 mg/kg·bw/day), MFL: low dose administration of miracle fruit (butanol extract, 500 mg/kg·bw/day), MFH: high dose administration of miracle fruit (butanol extract, 1000 mg/kg·bw/day).

**Table 3:** Effect of butanol extract from miracle fruit on serum CRE and BUN levels of hyperuricemia mice.

Groups	CRE	BUN
	mg/dL	
Normal	0.55 ± 0.06	19.6 ± 1.1
NC	0.65 ± 0.21	26.3 ± 3.6
PC	0.48 ± 0.14	22.4 ± 5.9
MFL	0.48 ± 0.07	22.5 ± 7.1
MFH	0.46 ± 0.13	22.4 ± 4.0

Each value is expressed as mean ± S.D. (*n* = 5). Value in a column with the different superscripts are significantly different (*p* < 0.05). Normal: blank, NC: negative control, PC: positive control (allopurinol, 10 mg/kg·bw/day), MFL: low dose administration of miracle fruit (butanol extract, 500 mg/kg·bw/day), MFH: high dose administration of miracle fruit (butanol extract, 1000 mg/kg·bw/day). CRE: creatinine; BUN: blood urea nitrogen.

As shown in Table 4, i.p. injection of oxonic acid potassium salt (negative control) significantly elevated hepatic xanthine oxidase activity in ICR mice, as compared to the normal group. However, HFL and allopurinol markedly attenuated these effects. Similarly, serum uric acid levels were markedly increased by oxonic acid potassium salt, and were suppressed by HFL or allopurinol. Interestingly, the effect of HFL was similar to allopurinol.

**Table 4:** Effect of butanol extract from miracle fruit on hepatic xanthine oxidase activity of hyperuricemia mice.

Groups	Xanthine Oxidase Activity
	(nmol/min/mg/protein)
Normal	1.07 ± 0.31 ^ab^
NC	1.35 ± 0.40 ^a^
PC	0.75 ± 0.11 ^b^
MFL	1.02 ± 0.27 ^ab^
MFH	0.82 ± 0.30 ^b^

Each value is expressed as mean ± S.D. (*n* = 5). Value in a column with the different superscripts are significantly different (*p* < 0.05). Normal: blank, NC: negative control, PC: positive control (allopurinol, 10 mg/kg·bw/day), MFL: low dose administration of miracle fruit (butanol extract, 500 mg/kg·bw/day), MFH: high dose administration of miracle fruit (butanol extract, 1000 mg/kg·bw/day).

**Figure 5:**
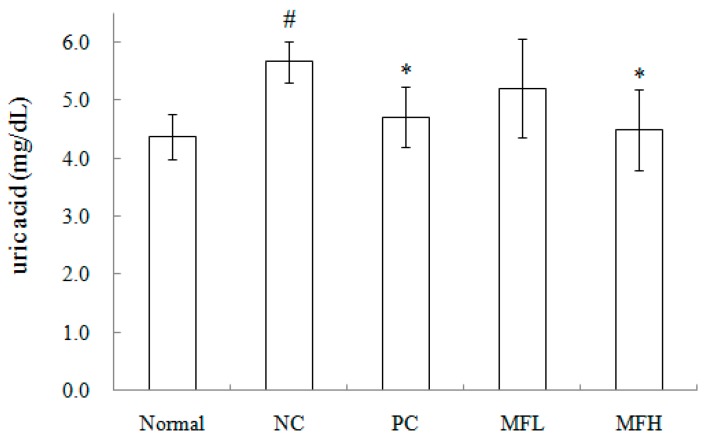
Effect of butanol extract from miracle fruit on the level of serum uric acid in hyperuricemia mice. Each value is expressed as mean ± S.D. (*n* = 5). Value in a column with the different superscripts are significantly different (*p* < 0.05). Normal: blank, NC: negative control, PC: positive control (allopurinol, 10 mg/kg·bw/day), MFL: low dose administration of miracle fruit (butanol extract, 500 mg/kg·bw/day), MFH: high dose administration of miracle fruit (butanol extract, 1000 mg/kg·bw/day). # Compared with Normal; * Compared with NC.

We hypothesized that the anti-gouty arthritis effects of miracle fruit are caused by antioxidants. Our results indicate that the water, butanol, EA, and hexane fractions of miracle fruit can inhibit xanthine oxidase activity *in vitro* (Figure 1 and Figure 2) and in MSU-treated RAW264.7 macrophages (Supplemental Figure S2). Moreover, the EA fraction attenuated uric acid accumulation (Figure 5) and hepatic xanthine oxidase activity (Table 4) in oxonic acid potassium salt-treated ICR mice. The pathway by which MSU induces inflammasome activation is well described [19,20]. This study investigated the potential capacity of miracle fruit to inhibit hyperuricaemia *in vitro* and *in vivo*. However, the mechanism by which miracle fruit extract exerts its effects remains unclear. Thus, the potential pathways and major compounds in miracle fruit extract regulating hyperuricaemia need to be investigated in the future.

## 3. Materials and Methods

### 3.1. Chemicals

1,1-diphenyl-2-pichryl hydrazyl (DPPH), 2,2′-azino-bis (3-ethylbenzthiasoline-6-sulfonic acid) (ABTS), butylated hydroxyanisole (BHA), peroxidase, potassium hexacyano-ferrate (III), Folin–Ciocalteu’s reagent, *p*-nitroblue tetrazolium chloride (NBT), 2′,7′-dichlorodihydrofluorescein diacetate (DCHF-DA), and iron(III) chloride were purchased form Sigma (St. Louis, MO, USA). Potassium dihydrogen phosphate (KH_2_PO_4_), di-Potassium hydrogen phosphate (K_2_HPO_4_) and di-sodium hydrogen phosphate (Na_2_HPO_4_) were purchased from Merk (Darmstadt, Germany)*.* Serum uric acid was determined by ELISA kit (Fortress diagnostics). CRE: creatinine (CRE) and blood urea nitrogen (BUN) ELISA kits were purchased from Randox Laboratories Ltd. (Antrim, UK).

### 3.2. Sample Preparation

Miracle fruit was vacuum-dried and ground to a powder. The 25 g of miracle fruit powder (MFP) was extracted by 250 mL of water for 40 min, and the extraction solution was filtered, and the filtered liquid was vacuum-concentrated and stored at −20 °C, it is referred to as miracle fruit-water extract (MFWE). On the other hand, MFP was extracted with butanol, and this butanol extracts were partied by water (water fraction) after filtration. This water fraction was carried out the partition with butanol (butanol fraction), hexane (hexane fraction), and ethyl acetate (EA fraction).

### 3.3. Antioxidation

The DPPH activity was measured by the method of Shimada *et al.* [21]. Briefly, a sample and a methanolic solution of DPPH were mixed and kept in the dark for 60 min. The absorbance of the reaction mixture at 517 nm was determined. The antioxidant capacity was determined by the method of Miller and Rice-Evans and Arnao *et al.* [22,23]. Peroxidase, H_2_O_2_, ABTS, and distilled water were mixed and stored in the dark for 1 h at 25 °C. A sample was subsequently added and the absorbance at 734 nm was determined. According to the method of Yen and Chen [24], aliquots of 0.5 mL extracts after appropriate dilution was mixed thoroughly with 0.5 mL of phosphate buffer (0.2 M, pH 6.6) and 0.5 mL of potassium ferricyanide solution. All mixtures were incubated in a water bath at 50 °C for 20 min and then rapidly cooled in an ice bath. Into each tube, 0.5 mL of 10% (*w*/*v*) trichloroacetic acid solution was added and thoroughly mixed by vortexing. After centrifugation of the tubes (1000*× g* at 20 °C), 1.0 mL of the supernatant was withdrawn and mixed with 1.0 mL water and 0.2 mL of 0.1% (*w*/*v*) ferric chloride solution. Then, the mixed solution was incubated at the ambient temperature (25–28 °C) without light exposure for 10 min and followed by absorbance determination at 700 nm. The total flavonoid content was determined using the method described by Abu *et al* [25]. Briefly, 0.5 mL of the extract was mixed with 2.25 mL of distilled water in a test tube followed by addition of 0.15 mL of 5 % NaNO_2_ solution. After 6 min, 0.3 mL (10%) AlCl_3_ solution was added and allowed to stand for another 5 min before 1.0 mL of 1 M NaOH was added. The absorbance of the reaction mixture at 510 nm was determined. Sample was dissolved in deionized water and the concentration of total phenolic compounds was measured by Folin–Ciocalteu’s reagent. Sample solution (100 μL), Folin–Ciocalteu’s reagent(500 μL), sodium carbonate (400 μL, 75 g/L) and deionised water(5 mL) were mixed thoroughly and kept at room temperature for 30 min before the absorbance at 760 nm was measured. Total phenolic content was determined using gallic acid as the standard [26].

### 3.4. Inhibition of Xanthine Oxidase Activity in Vitro

The 50 μL of sample was mixed with 35 μL of PBS (70 mM, pH 7.5) and reacted with 30 μL of xanthine oxidase (0.01 U/mL) at 25 °C for 15 min. Subsequently, the 60 μL of xanthine (150 μM) was added and reacted for 30 min, this reaction was stopped by HCL (1 N). The absorbance was measured at 290 nm, and allpourinol was used as the positive control [27]. Inhibitory activity (%) = (1 − A_sample_ at 290 nm/A_blank_ at 290 nm) × 100.

### 3.5. Cell Culture

The RAW264.7 cell line (BCRC 60001) was obtained from Bioresource Collection and Research Center (BCRC; Food Industry Research and Development Institute, Hsinchu, Taiwan). RAW264.7 cell line was cultured in Dulbecco’s Modified Eagle Medium (DMEM, Gibco, Grand Island, NY, USA) supplemented with 10% fetal bovine serum (FBS, Gibco), 100 IU/mL penicillin and 100 μg/mL streptomycin (DMEM-FBS) and maintained at 37 °C in a 5% CO_2_ humidified incubator. RAW264.7 cells were treated with various concentrations of samples and MSU (2.5 mg/mL) for 24 h. The medium was removed to collect cell pellets for measurement of xanthine oxidase according to Orallo *et al.* [28]. The concentration of uric acid was 1.22 × 10^4^ (mmol/mL)/cm^3^. Thus, the formula was: xanthine oxidase activity (U/mg protein) = ((dA_290_ nm/min) × 1000 × time)/(1.22 × 10^4^ × sample volume × protein concentration).

### 3.6. Animal Model

Male ICR mice (5 weeks old) (BioLASCO, Taiwan Co., Ltd., Yilan, Taiwan) were housed in individual plastic cages and subjected to a 12 h light-dark cycle with 60% relative humidity at 25 ± 2 °C. The animals were given free access to regular rodent chow diet and water for 1 week to adapt to the new environment. The experiments were carried out in a qualified animal breeding room in the animal center at our institute (Protocol complied with guidelines described in the “Animal Protection Law”, amended on 17 January 2001 Hua-Zong-(1)-Yi-Tzi-9000007530, Council of Agriculture, Executive Yuan, Taiwan). The animal study was approved by Institutional Animal Care and Use Committee (IACUC) of National Chiayi University. Animals were randomly divided into 5 groups (*n* = 5), including (1) blank (PBS i.p. injection + PBS oral administration); (2) NC: negative control (i.p. injection with oxonic acid potassium salt; 250 mg/kg·bw for 7 days + PBS oral administration); (3) PC: i.p. injection with oxonic acid potassium salt + positive control (allopurinol, 10 mg/kg·bw/day; oral administration); (4) i.p. injection with oxonic acid potassium salt + MFL: low dosage of miracle fruit-butanol extract, 500 mg/kg·bw/day; oral administration); (5) i.p. injection with oxonic acid potassium salt injection + MFH: high dosage of miracle fruit-butanol extract, 1000 mg/kg·bw/day; oral administration). After administration over 7 days, animals were sacrificed and blood was collected and stored at −80 °C until the following experiments.

### 3.7. Measurement of Hepatic Xanthine Oxidase Activity

The hepatic xanthine oxidase activity was determined spectrophotometrically using standard diagnostic kits purchased from Jiancheng Biotech (Nanjing, China). The xanthine oxidase activity is expressed as nmol/min/mg protein.

### 3.8. Measurement of Oxidative Stress

The level of oxidative stress was monitored by the measurement of reactive oxygen species. Collected cells were suspended in 500 μL of PBS and mixed with 10 μM (final concentration) DCFH-DA to incubate for 20 min at 37 °C. The cells were washed three times with PBS to remove redundant DCFH-DA. The cell pellet was mixed with 500 μL of PBS, and the oxidative stress was assayed by ELISA reader [29].

### 3.9. Statistical Analysis

The analysis of variance was used to evaluate the significance of the differences between factors and levels. Comparison of the means was carried out by employing a Student’s *t*-test to identify which groups were significantly different from other groups. The least significant difference was *p* < 0.05.

## 4. Conclusions

Recently, a large of amount of antioxidants including polyphenolic compounds and flavonoids have been found in several berries [30,31]. In addition, other studies also found that antioxidants isolated from strawberries could show the inhibitory effects of inflammation and oxidative stress [32,33]. Currently, MFP, MFWE, and various fractions from miracle fruit have antioxidant effects and can attenuate hyperuricaemia *in vitro* and *in vivo*. Taken together, our results suggest that butanol extracts from miracle fruit may be an effective treatment for acute gouty arthritis and could be developed as a health food.

## Supplementary Materials

Supplementary materials can be accessed at: http://www.mdpi.com/1420-3049/21/2/140/s1.

## Abbreviations

ABTS: 2,2′-azino-bis (3-ethylbenzthiasoline-6-sulfonic acid)
BHA: butylated hydroxyanisole
BUN: blood urea nitrogen
CRE: creatinine
DCHF-DA: 2′,7′-dichlorodihydrofluorescein diacetate
DPPH: 1,1-diphenyl-2-pichryl hydrazyl
MFP: miracle fruit powder
MFWE: miracle fruit water extract
MSU: monosodiumurate
NBT: *p*-nitroblue tetrazolium chloride
NLRP3: nucleotide-binding oligomerization domain-like receptor pyrindomain containing 3
ROS: oxygen species
