# Supplementary material for: Miracle Fruit (Synsepalum dulcificum) Exhibits as a Novel Anti-Hyperuricaemia Agent
Source: Molecules. 2016 Jan 26;21(2):140. doi: 10.3390/molecules21020140 (PMC6274476; doi:10.3390/molecules21020140)
Supplement: Supplementary file 1 [file molecules-21-00140-s001.pdf]

Supplementary Material: Miracle Fruit (*Synsepalum dulcificum*) Exhibits as a Novel Anti-Hyperuricaemia Agent

Yeu-Ching Shi, Kai-Sian Lin, Yi-Fen Jhai,Bao-Hong Lee,Yifan Han, Zhibin Cui, Wei-Hsuan Hsu and She-Ching Wu

Talbe S1. The antioxidant levels of miracle fruit powder (MFP) and miracle fruit-water extract (MFWE).

Sample	Total Phenolics	Total Flavonoids	Total Anthocyanins
		(mg/g dry weight)	
MFP	11.63 ± 0.16	0.79 ± 0.05	0.21 ± 0.04
MFWE	7.44 ± 0.33	0.55 ± 0.03	0.17 ± 0.12

Each value is expressed as mean ± S.D. (n = 3).

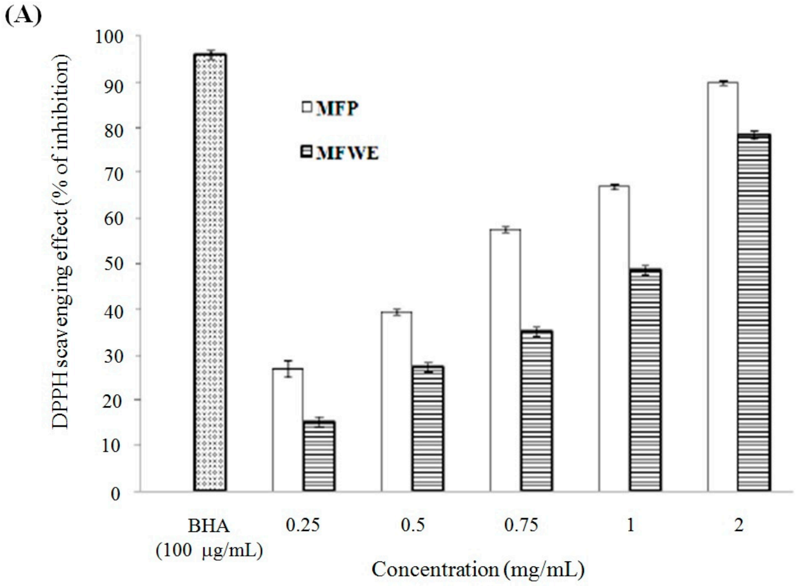

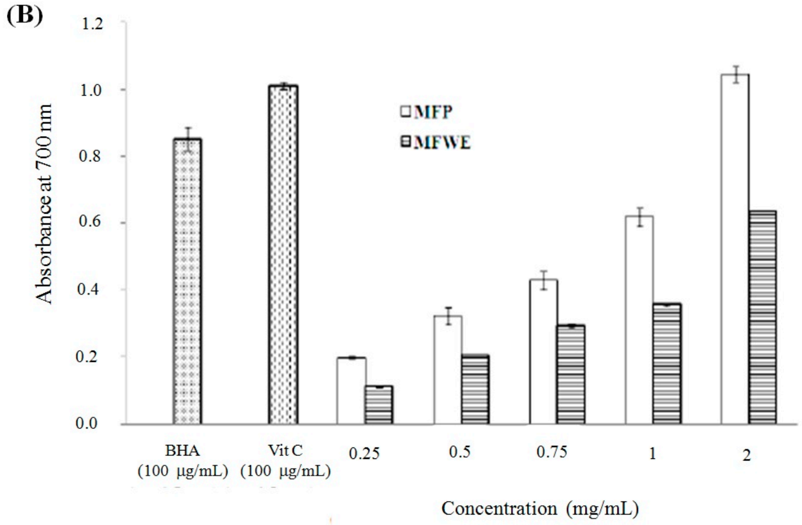

Figure S1. *Cont.*

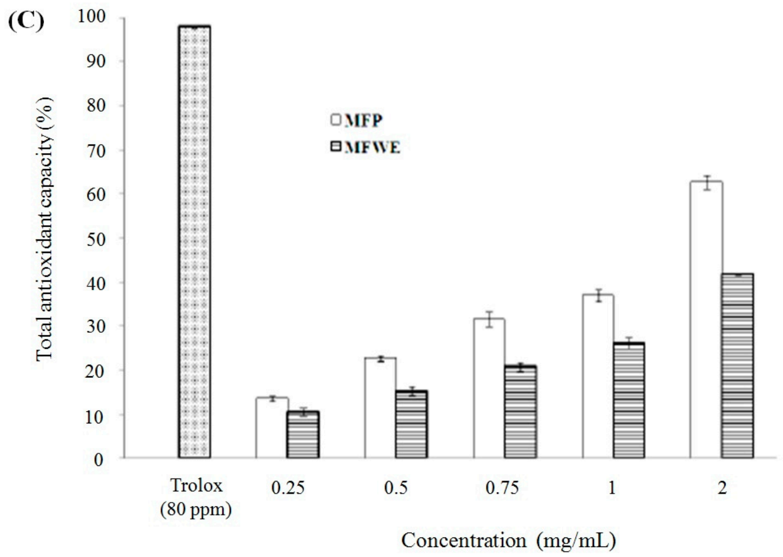

Figure S1. The effects ofmiracle fruit powder (MFP) and miracle fruit-water extract (MFWE) on (**A**) DPPH radical scavenging capacity; (**B**) reducing power; and (**C**) TEAC activity. Each value is expressed as mean ± S.D. (n = 3).

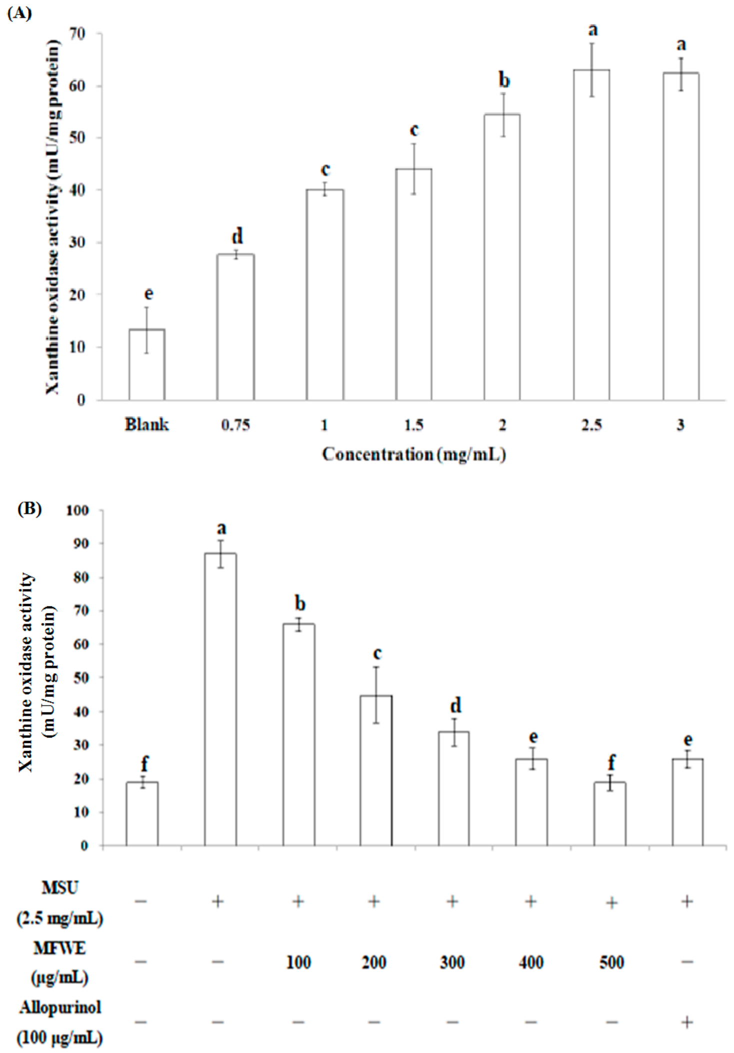

Figure S2. The inhibitory effects ofmiracle fruit-water extract (MFWE)on xanthine oxidase activity *in vitro* (**A**); and in MSU-treated RAW264.7 cells (**B**). Each value is expressed as mean ±S.D. (n = 3).
